# Nightly variations in sleep quality and next-day cognitive performance: an in-home study in healthy older adults

**DOI:** 10.3389/fnagi.2026.1714063

**Published:** 2026-03-09

**Authors:** Mary Brooks, Randa El Chami, Hugo R. Jourde, Marie-Anick Savard, Emily B. J. Coffey

**Affiliations:** 1 Department of Psychology, Concordia University, Montreal, QC, Canada; 2 EngAGE: Center for Research on Aging, Montreal, QC, Canada; 3 Montreal Neurological Institute, McGill University, Montreal, QC, Canada

**Keywords:** aging, cognition, sleep quality, sleep variability, wearables

## Abstract

**Introduction:**

Sleep quality is often thought to be a key determinant of cognitive performance, particularly in older adults who experience age-related changes in sleep architecture. However, the extent to which nightly variations in sleep quality impact next-day cognitive performance remains unclear—in part because it has only recently become practical to measure sleep over multiple nights.

**Methods:**

In this study, we used an in-home wearable electroencephalography (EEG) device to monitor sleep patterns over ~10 nights in 17 healthy older adults, assessing metrics of sleep quality such as wake after sleep onset and the density of slow oscillations and sleep spindles. Next-day cognitive performance was evaluated using two computerized neuropsychological tasks measuring executive functions (inhibition and cognitive flexibility), and their relationships to sleep metrics were explored.

**Results:**

Although participants placed the EEG device themselves, a high proportion of sleep data was usable (~71%), and clear nightly variations in sleep quality were captured. Sleep recordings showed considerable variability in sleep quality metrics across nights, with large inter-individual differences. However, we found no effects of either macro- or microarchitectural sleep metrics on executive task outcomes the following day.

**Discussion:**

These results do not rule out the possibility that some aspects of cognitive performance may be affected by daily fluctuations in sleep quality; however, they suggest that inhibition and cognitive flexibility, which underlie reasoning and problem solving, may be relatively resilient to nightly sleep variability in older adults. The findings also demonstrate the feasibility of using emerging portable devices to extend sleep studies at home and over multiple nights in older adults, while providing variance estimates and effect sizes to guide power and sample size planning for future studies.

## 1 Introduction

As populations worldwide age, understanding the relationship between age, cognition, and sleep is becoming increasingly important. Both cognitive abilities and sleep patterns undergo significant changes as we age, and growing evidence suggests these changes may be interconnected. Aging is accompanied by declines in some aspects of cognition. For example, compared to young adults, older adults show worse reaction times and motor control (Vallesi et al., 2021), and perform worse on measures of reasoning and verbal fluency (Sánchez-Izquierdo and Fernández-Ballesteros, 2021). Executive functions, which are top-down higher-order mental processes involved in purposeful, goal-directed, and future-oriented behaviors (Suchy, 2009), are particularly affected. Inhibition and cognitive flexibility are two executive functions of particular interest in aging as they affect independence and everyday activities (Diamond, 2013; McAlister and Schmitter-Edgecombe, 2016). Inhibition encompasses self-control, selective attention, and response suppression (Guarino et al., 2020). Older adults show increased commission errors (responding when action should be withheld; Boywitt et al., 2015) and greater interference effects on tasks requiring suppression of irrelevant information (Bialystok et al., 2004). Cognitive flexibility (i.e., the ability to adapt to environmental changes) also declines, with older adults showing higher task-switching costs (Hakun et al., 2015).

With age, sleep changes significantly. In general, older adults spend less time in deep sleep (non-rapid eye movement sleep stage 3; N3) and have more fragmented sleep, indicated by more frequent arousals and increased “wake after sleep onset” (WASO) duration. Many older adults also subjectively report worse and more variable sleep quality compared to young adults (Shoji et al., 2015). Poor subjective sleep can negatively affect quality of life and has been associated with a decline in overall wellbeing and mental health, and a higher risk of dementia (Reid et al., 2006; Okun et al., 2011; Qin et al., 2023). Numerous studies have linked age-related changes in sleep quality to poorer cognition in older adults, including impaired motor control, reduced attention, and slower reaction times (McCrae et al., 2012; Nebes et al., 2009; Blackwell et al., 2011; Spira et al., 2017; Miyata et al., 2013). Consistent with these findings, a study by Wilckens et al. (2014) reported that higher average WASO measured over a 1-week period was associated with higher “task switching” costs in both young and older adults. There is also growing evidence that within-participant sleep variability–meaning greater fluctuations in sleep measures across nights within the same individual–may also correlate with worse cognitive outcomes. For example, greater within-participant variability in sleep duration was linked to increased risk of cognitive decline in older adults (Keil et al., 2023), and greater intraindividual variability in WASO was associated with poorer attention (Kuan et al., 2022). Taken together, these findings suggest that both the overall decline in sleep quality with age and the consistency of sleep from night to night may influence cognitive functioning in older adults.

Questionnaires and actigraphy have been widely used to measure sleep quality; however, neither allows researchers to capture neurophysiological information about sleep depth and structure. Of studies measuring sleep quality using electroencephalography (EEG), which facilitates a deeper investigation of sleep neurophysiology, few studies have focused on the finer-grained aspects of sleep physiology or sleep “microarchitecture.” Sleep spindles and slow oscillations are two microarchitectural features that change considerably with age. Sleep spindles are transient bursts (0.5–2 s) of activity between 11 and 16 Hz that are hallmarks of stage 2 sleep and have been associated with memory consolidation, with spindle density (i.e., number of spindles per minute), amplitude, and duration all reduced in older adults (Ulrich, 2016; Clawson et al., 2016; Purcell et al., 2017; Martin et al., 2013). Slow oscillations (SOs) are high amplitude, low frequency (< 1 Hz) events that occur mainly during deep sleep (N3). They are characterized by synchronous neuronal activity that represents widespread fluctuations in cortical excitability. The coordination of cortical and subcortical regions are thought to be involved in memory consolidation and neuronal plasticity (Massimini et al., 2004). Similarly to spindles, SO density, amplitude, and peak-to-trough slope tend to be reduced in older adults (Rosinvil et al., 2021). Given their role in memory consolidation and neuronal plasticity, age-related changes in spindles and SOs may contribute to cognitive changes observed in aging.

Although most studies to date examining the relationships between microarchitectural features and cognition have focused on memory processes (see Scullin and Bliwise, 2015 for a comprehensive review), there is some evidence that these features are associated with cognitive functions more generally. For example, spindle density has been associated with better sustained attention (Lafortune et al., 2014), inhibition, and cognitive flexibility (Guadagni et al., 2021). Slow oscillations are thought to promote neuronal plasticity through synaptic homeostasis, potentially improving learning and attention during wakefulness (Tononi and Cirelli, 2014). Supporting this relationship, acoustic stimulation studies have shown that enhancing slow oscillations can improve attention and alertness following sleep restriction in young adults (Diep et al., 2020). These findings suggest spindles and slow oscillations may underlie observed links between sleep measures and executive functions, including attention and inhibition.

Poor sleep quality has been associated with worse cognitive performance in older adults. However, research on the effects of nightly sleep quality on daily cognitive performance using objective, microarchitectural EEG measures remains limited. Multi-night studies are necessary to obtain stable measures of sleep quality in a naturalistic setting, given first-night effects (i.e., poorer sleep the first night because of changes to environmental conditions such as sleeping in a sleep laboratory or with equipment), and natural sleep variability (Newell et al., 2012), yet few studies have reported the variance components necessary for appropriate sample size calculations in this population. Previous multi-night studies in older adults have mostly relied on subjective questionnaire-based measures or actigraphy. Further exploration of how specific sleep macro- and microarchitecture relates to cognition across multiple nights is needed to clarify their influence on behavior. To address this gap, we conducted an in-home study in which older adults wore a portable EEG device for 2 weeks and completed cognitive tasks each morning to assess how nightly sleep quality affects next-day cognitive performance.

The first objective of this study was to determine the feasibility of multi-night in-home EEG recordings in older adults. The second objective was to characterize variability in sleep quality using objective sleep measures (i.e., WASO, deep sleep duration, spindles, and slow oscillations) over a two-week period and to examine the relationship between average sleep quality and cognitive performance (i.e., inhibition and cognitive flexibility). Additionally, we explored whether greater within-participant sleep variability (i.e., using standard deviation as a measure) was associated with poorer cognitive performance. The final objective of the study was to explore the effect of nightly sleep quality on next-day cognitive performance. We expected that older adults would be able to record EEG data with similar data loss rates to those in recent studies using in-home devices. We hypothesized that poorer average sleep quality (higher WASO, less deep sleep, lower spindle and slow oscillation density) and greater variability across nights would be associated with worse performance on both cognitive tasks (slower reaction times and higher performance costs on incongruent and rule switching trials). Concerning nightly fluctuations in sleep quality, we hypothesized that lower sleep spindle and slow oscillation density and higher WASO on a particular night would be associated with worse next-day performance on the two cognitive tasks. This study design allowed us to investigate how nightly variation in sleep quality relates to next-day cognitive performance in older adults—capturing sleep in participants' typical home environments so as to reflect their natural sleep patterns.

## 2 Materials and methods

### 2.1 Participants

Twenty healthy older adults between the ages of 60 and 75 participated in the study. Our sample reflects relatively younger older adults, but should still capture the effects of interest since changes to cognition and sleep tend to occur around 60 years old (Vitiello, 2006; Scullin and Bliwise, 2015). Based on self-report, participants had no psychiatric or sleep disorders, and were not taking any medications targeting or which are known to affect sleep. They also had normal or corrected-to-normal hearing and eyesight and had not changed time zones in the 6 weeks preceding the experiment. Participants were required to own or have access to a computer and be comfortable using technology to participate. Participants were recruited from the local Montréal area, signed an informed consent form before participating in the study, and were compensated for their time. This study was approved by Concordia University's Human Research Ethics Committee (30017434).

### 2.2 Experimental design

**Figure 1:**
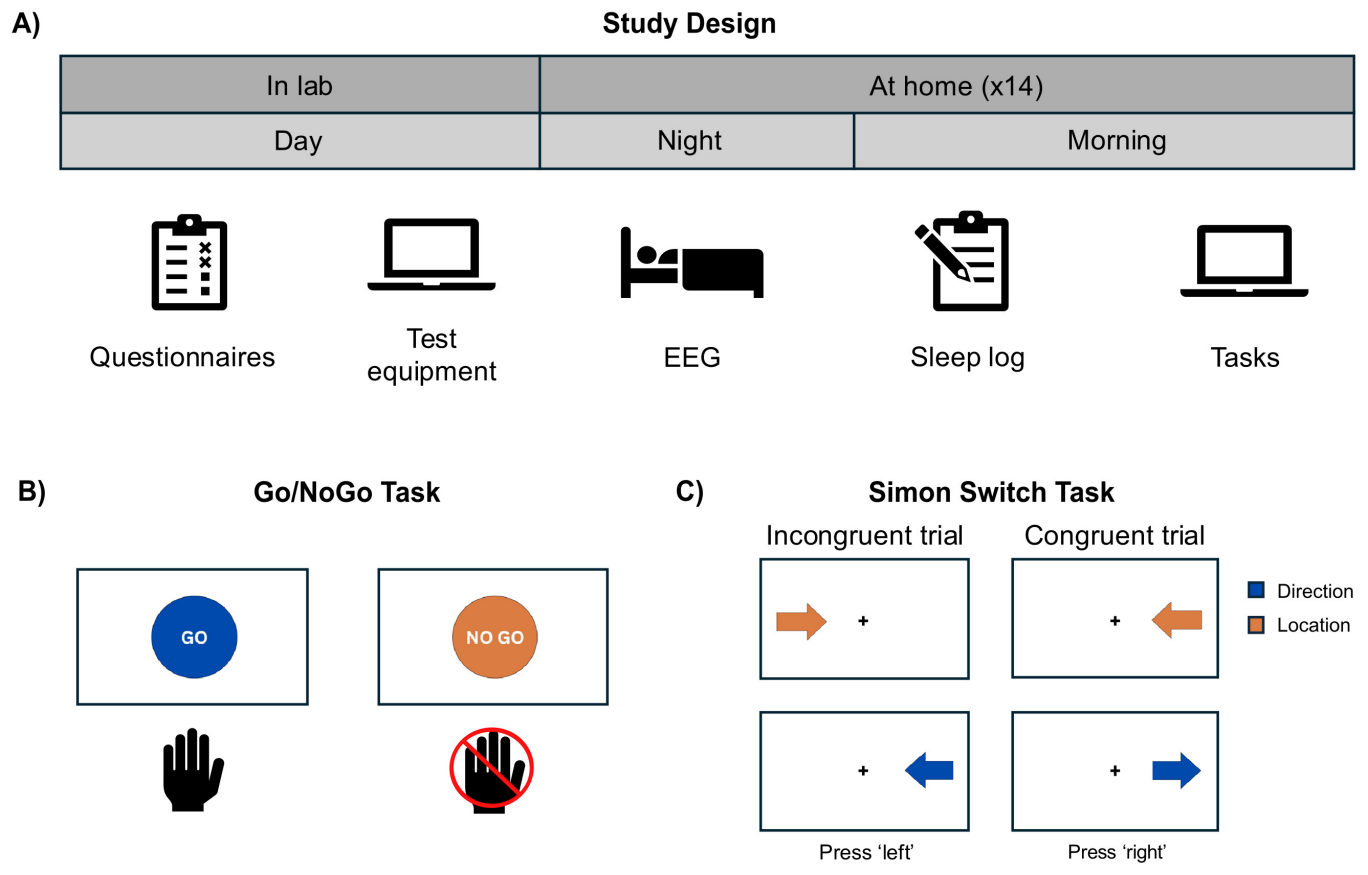
Study design and task overview. **(A)** Study protocol: participants completed questionnaires and were familiarized with the equipment and cognitive tasks in the lab. At home, participants wore an EEG device overnight, and completed a sleep log and the two cognitive tasks the following morning (~14 nights). **(B)** Schematic illustrating the Go/NoGo Task. Participants were asked to respond with a button press to “go” and inhibit their response when presented with “no go.” Reaction time and accuracy on the “go” trials were assessed. Commission error, or responding on a “no go” trial, was also measured. **(C)** Schematic illustration of the Simon switch task. Blue arrow symbols cued the participants to respond with a key press to indicate the direction the arrow was pointing, whereas orange arrows instructed participants to instead respond to the location of the arrow on the screen. Random presentation of conditions across trials allow us to measure the performance cost of incongruency (e.g., arrow pointing to the right but on the left side of the screen) and of rule-switching [i.e., changing from the arrow direction (blue) to arrow location (orange) rule]. Performance was measured using reaction time and accuracy (reaction time was our main measure of interest).

The study design is illustrated in Figure 1. Potential participants were screened by telephone using a standardized script. During the laboratory session, which lasted less than 2 hours, participants completed questionnaires about their general demographic and health information, and completed other questionnaires described below. Participants were instructed on how to correctly position the wireless headband-mounted EEG device designed for monitoring neurophysiological activity, how to test its connection with its smartphone application, and how to check signal quality. They were also familiarized with the tasks and provided with a printed manual containing step-by-step instructions for home reference.

The in-home portion of the study was completed over 14 nights. During the night, participants wore the EEG headband to measure electroencephalography during sleep (see below). In the morning, participants completed a daily sleep log as well as two self-administered computerized online cognitive tasks between 30 minutes and 4 hours after waking up. Although flexibility was permitted to encourage retention, participants were encouraged to complete the tasks about the same time each day. On average, participants completed the tasks around 9:00 a.m. (SD = 2.0 h). The order of tasks alternated every day. Completion of tasks and of the sleep log took approximately 15 minutes. Participants were encouraged to contact the experimenter by telephone or email if an issue arose with the equipment or tasks.

### 2.3 Questionnaires

Questionnaires were used to characterize the sample in terms of their levels of depression and anxiety, cognitive functioning, sleep quality, and chronotype. The Beck Anxiety Inventory (Beck et al., 1988) and Beck Depression Inventory-II (Beck et al., 1996) were used to measure levels of anxiety and depression. Both self-report questionnaires contain 21 items (each item scored from 0 to 3 on a 4-point Likert). Total scores range from 0 to 63 with higher scores indicating more severe depression. The Montreal Cognitive Assessment (MoCA) was used to measure cognitive functioning. This questionnaire is a one-page paper-and-pencil test commonly used to screen participants for basic cognitive functioning, including short-term memory, language, and executive functions (Nasreddine et al., 2005). A score of 26 or higher is indicative of normal cognitive functioning, while a lower score may indicate cognitive impairment. The Pittsburgh Sleep Quality Index (PSQI; Buysse et al., 1989) was used to capture overall subjective sleep quality (e.g., duration, disturbances, amount of time to fall asleep) over the past month. The Morningness-Eveningness Questionnaire (MEQ) was used to assess participants' natural preference for being active or asleep during certain times of the day (Horne and Ostberg, 1976). The total score ranges from 16 to 86, with higher scores indicating a greater preference for mornings and lower scores indicating a greater preference for evenings. A custom daily sleep log was used to measure sleep characteristics and current state. This short questionnaire consisted of eight questions. Participants were asked to rate their current state of mind, including their mood, how tired, stressed, sick, and distracted they felt using a 5-point Likert scale. Participants were also asked their bedtime and wake time.

### 2.4 Cognitive tasks

Two computerized cognitive tasks assessing executive function were selected for their brevity and suitability for measuring variability across multiple sessions. Their randomized trial presentation and lack of strategic elements reduce the risk of substantial learning effects, making them well suited for repeated assessments. Both tasks have been shown to be sensitive to sleep deprivation (Bratzke et al., 2012; Sagaspe et al., 2012). The tasks were coded in PsychoPy, version 2022.2.4 (Peirce, 2007) and hosted on Pavlovia^1^ for online data collection.

#### 2.4.1 Go/NoGo task

The Go/NoGo task (illustrated in Figure 1B) is designed to measure cognitive inhibition (Gomez et al., 2007) and sustained attention (Sagaspe et al., 2012). The task was adapted from an existing version (Vu, 2025). We changed the background from gray to black to increase the visibility of the stimuli through a higher stimulus-background contrast, as older adults exhibit well-documented declines in contrast sensitivity (Owsley, 2016). The task consisted of three blocks of 100 trials each, for a total of 300 trials (240 “go,” 60 “no go”). This 80/20 ratio was chosen to establish a strong prepotent response tendency, as infrequent “no go” trials have been shown to elicit more robust inhibitory demands (Wessel, 2018). Each trial lasted 1,000 ms. The “go” instruction was a blue circle with “go” in the center and the “no go” instruction was an orange circle with “no go” in the center. Participants were instructed to press the space bar as quickly and accurately as possible in response to the “go” instruction and to withhold their response when presented with the “no go” instruction. Reaction time was calculated by averaging the correct responses on “go” trials. Responses below 200 ms and above 900 ms were excluded as outliers, following Chuang et al. (2015). Inhibition response, or “commission error,” was calculated based on the number of incorrect responses, or a lack of inhibition, on “no go” trials relative to correct responses.

#### 2.4.2 Simon switch task

The Simon switch task involves shifting between different response rules based on different cues (Cohen et al., 2008; see Figure 1C for an illustration) and can be used to measure both interference resolution and cognitive flexibility. The task was adapted from an existing version (Zhang, 2025). The colors of the arrows were changed to blue and orange (originally red and green) to accommodate individuals with impaired color vision (red-green color-blindness being the most common form). In this task, participants were presented with arrows on the screen, with the color (blue/orange), location (left/right of the screen), and direction (pointing left/right) of the arrows changing for each trial. When the participant saw a blue arrow, they were instructed to indicate the direction in which the arrow was pointing no matter where the arrow appeared (i.e., “Direction Task”). When they saw an orange arrow, they were instructed to indicate where its location on the screen, no matter which direction the arrows pointed (i.e., “Location Task”). To respond, the participant pressed the “z” key on the keyboard if the response was “left” and the “m” key if the response was “right,” which on an English-language keyboard are located to the left and right of the keyboard. The task was divided into 3 blocks of 100 trials, each trial ending when the participant responded (or after 4,000 ms). Responses below 300 ms and above 3,000 ms were excluded as outliers, following Cohen et al. (2008). The Simon effect measures interference resolution and refers to the performance cost observed when spatial location and response direction are incongruent (i.e., when the location and direction of the arrow do not match), even though one of these cues is task-irrelevant (Simon, 1990). The Simon effect on reaction time was calculated by subtracting reaction time on congruent trials from reaction time on incongruent trials. The Simon effect on accuracy was calculated by subtracting the percentage of correct responses on incongruent trials from congruent trials. The switch effect measures cognitive flexibility and refers to the performance cost observed when participants must change between task rules. Specifically, it compares trials where the instruction changed from the previous trial (switch trials) vs. trials where the same instruction was repeated (non-switch trials), regardless of the specific color cue. The switch effect on reaction time was calculated by subtracting reaction time on non-switch trials from switch trials. The switch effect on accuracy was calculated by subtracting percent correct on switch trials from non-switch trials. The Simon and switch effect on reaction time were used as the main metrics of interest as indicated in Simon (1990).

### 2.5 Sleep recording

A portable, headband-style EEG device (the DREEM-2; Arnal et al., 2020) was used to measure sleep. The headband has five dry electrodes placed according to the 10–20 EEG system (F7, F8, Fpz, O1, and O2), yielding seven derivations (Fpz-O1, Fpz-O2, Fpz-F7, F8-F7, F7-O1, F8-O2, Fpz-F8; 250 Hz sampling rate with a 0.4–35 Hz bandpass filter). The electrodes are made of flexible silicone with soft protrusions that enable signal acquisition through hair. The device also records heart rate via pulse oximeter and movement/position via a 3D accelerometer. Although this montage lacks the central derivations (e.g., C3, C4) typically used in sleep polysomnography, previous studies have validated the DREEM headband and its automatic sleep staging algorithm against laboratory-grade PSG across several populations (Arnal et al., 2020; Berryhill et al., 2020; Guillot et al., 2020; Zambelli et al., 2022; Dogaheh and Moradi, 2020); sleep staging follows AASM guidelines (Iber, 2007). Additional technical specifications have been published previously (Debellemaniere et al., 2018). The headband was connected via Bluetooth to a smartphone running Dreem's “Alfin” application, which allowed participants to verify signal quality, initiate recordings, and track sessions. Participants were instructed to charge the headband each morning, which triggered automatic upload of recordings to a secure server for monitoring by the experimenter. Although the original study plan called for 30 participants, this was not possible due to commercial disruption (i.e., headsets and recording platform became unsupported mid-study due to a change of ownership). For this reason, we have had to abbreviate some of our explorations that would require larger sample sizes. Nonetheless, our main research questions can be addressed with the present sample, using generalized linear mixed models (see below). To minimize disruption in future work, we encourage the further development of open science, low-cost, community-supported projects for basic science applications (e.g., the Portiloop; Valenchon et al., 2022).

#### 2.5.1 Slow oscillation detection

**Figure 2:**
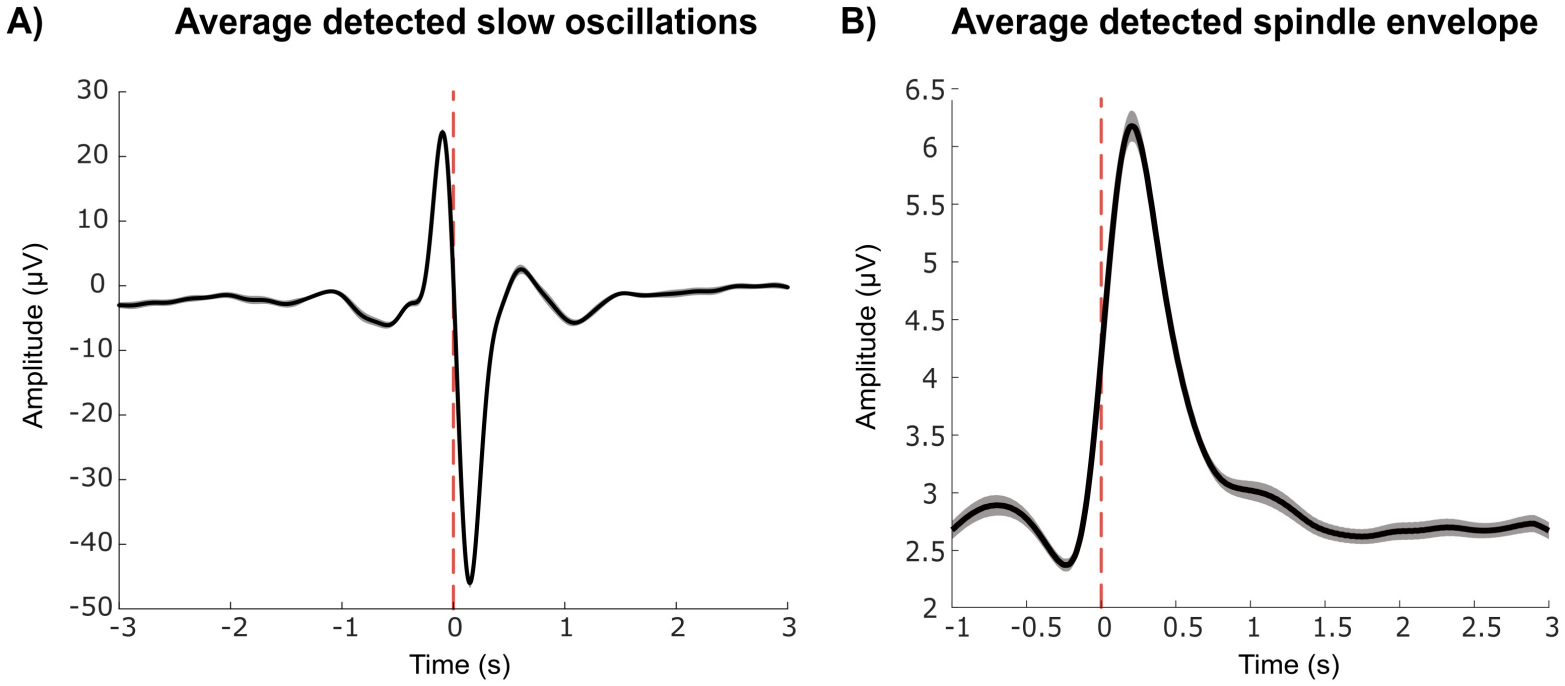
**(A)** Average slow oscillations (i.e., 0.5–4 Hz) and **(B)** spindle envelope (i.e., 11–16 Hz) across all participants, demonstrating that slow oscillations and spindles were successfully detected using in-home recordings. The dashed vertical red lines refer to the time of sleep event detection. Shaded areas refer to the standard error of the mean (SEM).

Slow oscillations (SOs) were automatically detected offline using the raw data provided by the DREEM-2 headband. The same channels and quality threshold used for spindle detection were used for SO detection. The detection was run on artifact-free N2 and N3 sleep. Using custom MATLAB (The MathWorks Inc, 2022) scripts, data were first filtered between 0.3 and 40 Hz by applying a 300th-order finite impulse response (FIR) filter using the “fir1” function in MATLAB (The MathWorks Inc, 2022) then the data were divided into 30 s epochs. Each epoch was demeaned to remove baseline offsets and epochs exceeding 200 μV were excluded. The data were subsequently bandpass filtered between 0.3 and 4 Hz. The following parameters were used for SO detection: negative deflection = 33 μV, peak-to-peak amplitude = 61 μV, minimum negative deflection = 125 ms; max negative deflection = 1,500 ms; maximum positive deflection = 1,000 ms; as described in Lafrenière et al. (2023). Averages of slow oscillation amplitude were examined for each participant (see Figure 2A for the group average). Additionally, a random subset of detections were visually inspected to ensure that a slow oscillation occurred. Slow oscillation density was defined as the number of slow oscillations divided by the number of minutes spent in N2 and N3 sleep. Slow oscillation amplitude was calculated by averaging the peak-to-peak amplitude per night and per participant.

#### 2.5.2 Spindle detection

Spindle detection was performed offline using the “A7” algorithm developed by Lacourse et al. (2019) on the raw EEG data. One of four channels was used depending on the channel with the best quality: F7-O1, F8-O2, Fpz-O1, Fpz-O2. DREEM provides the average channel quality (the percentage of scoreable EEG data across the night) for each electrode. We used a quality threshold of 50% to preserve as much usable data as possible. If all four channels had less than 50% quality, that night was discarded from the analysis. Detection was performed on artifact-free N2 and N3 sleep. The Lacourse algorithm originally uses four criteria: absolute power in the sigma frequency band (11–16 Hz; the range associated with sleep spindles), relative sigma power, and both correlation and covariance between the sigma band-filtered signal and the broadband EEG signal (0.4–30 Hz) on a sliding window of 0.3 s (0.1 s between consecutive windows). Absolute spindle power was omitted in our implementation because we used a different electrode montage which may have different noise characteristics as compared with the EEG signal on which the detection algorithm was developed and validated. Otherwise, default parameters were used. Spindles were defined as events lasting between 0.5 and 2.5 s, similar thresholds have been used in other studies in older adults, for example Orlando et al. (2024) used 0.5 and 3 s, Purcell et al. (2017) used 0.3–3 s, and Poirson et al. (2024) used 0.5–2 s. The average spindle envelope for each participant was examined to ensure that events of interest were captured (see Figure 2B for the group average). Additionally, random spindle events were visually inspected to ensure that a spindle was detected. Spindle density was calculated using the number of spindles divided by the number of minutes spent in N2 and N3 sleep. Spindle amplitude was calculated using the average sigma envelope per night and per participant.

### 2.6 Statistical analyses

To evaluate the feasibility of multi-night EEG recordings in older adults, we assessed EEG data quality across nights for each participant. The number of usable nights was determined by removing data with less than 50% channel quality. Missing or incomplete data (e.g., missing cognitive or sleep data) were not included in the analyses. Three participants were excluded for having less than four nights of good recording quality, leaving 17 participants for the analyses. One participant accidentally recorded an additional night at the end of the study, and this night was included in the analysis. For one participant, nine nights were lost due to a connectivity issue between the headband and the smartphone; the remaining five nights were included. To verify that wearing the device did not disrupt sleep, a generalized linear mixed model (GLMM) with WASO as the outcome and night as the predictor was conducted. GLMMs will be described in further detail below. To summarize sleep quality and its variability across nights in our sample, we computed means and standard deviations. Means were used to characterize between-participant sleep quality variability, and the standard deviation of sleep metrics were used to characterize within-participant sleep quality variability. To illustrate sleep quality variability across and within-participants, we produced boxplots of spindle and slow oscillation density, WASO, and minutes spent in N3 (deep sleep), our main variables of interest. These metrics were chosen on the basis that previous research shows their relationship to cognitive functioning in older adults (Kuan et al., 2022; Lafortune et al., 2014; Wilckens et al., 2016).

We performed correlational analyses to investigate the relationship between average sleep quality and cognitive performance on the two tasks. Two-tailed Pearson correlations were performed between mean sleep metrics and mean cognitive variables across nights. Inspection of the mean metrics using Q-Q plots and histograms of the variables revealed that normality was not violated. Additionally, we conducted exploratory correlational analyses to assess the relationship between within-participant sleep variability and cognitive performance. Standard deviations of sleep measures and means of cognitive performance were used. Inspection of histograms for standard deviations revealed non-normal distributions; nonparametric Spearman correlations were performed in this case. All correlation *p*-values were corrected for multiple comparisons using the false discovery rate (FDR) correction unless specified.

We used generalized linear mixed models (GLMMs) to investigate the role of nightly sleep quality on next-day cognitive performance (i.e., mean reaction time, commission error, mean switch effect on reaction time, and mean Simon effect on reaction time). GLMMs were chosen for their ability to handle small datasets and their flexibility with respect to distribution shape (Bolker, 2015). GLMMs allow us to measure both fixed and random effects. Random effects account for variability among the levels of the predictor, whereas fixed effects estimate the average effect. For each model, we included participant as a random effect to account for individual variability and sleep and cognitive variables as fixed effects. Inspection of the frequency distribution using histograms and inspection of Q-Q plots of the residuals revealed non-normal distributions for commission error, spindle density, and slow oscillation density. Based on visual inspection, one outlier was identified as substantially higher than the rest of the distribution for slow oscillation density and was removed. Additionally, one participant was missing reaction time and commission error values for one night, and this day was excluded from the analysis. The variable “commission error” is expressed as a percentage and was divided by 100 to rescale the data between 0 and 1. The data were highly positively skewed and transformations did not alter the distribution; as a result the rescaled data were retained as proportion values. The commission error model was then specified as a beta distribution (set between 0 and 1). All GLMM and correlation analyses were performed in R version 4.4.0, for mixed models, we used the glmmTMB package (Brooks et al., 2017). To assess the significance of the model terms, the emmeans package was used (Lenth, 2016). Boxplots were created using JASP (Version 0.19.3; JASP Team, 2025).

Intraclass correlation coefficients (ICC) were calculated for all GLMMs. The ICCs can inform future studies as regards the required sample size and number of measurements needed per participant (Mondal et al., 2024), addressing a gap in the literature for multi-night sleep-cognition studies in older adults. The ICC represents the proportion of total variance on our outcome measures (i.e., reaction time, commission error, the Simon effect and switch effect) that can be attributed to between-participant differences as compared to within-participant variability. The ICC is calculated using the between- and within-participant variance model components as follows: ICC = σ^2^ between-participants/(σ^2^ between-participants + σ^2^ within-participants). A high ICC would indicate strong between-participant differences as compared to within-participant differences account for the variance in the outcome. For each model, the ICC was calculated and the 95% confidence interval was obtained using parametric bootstrapping. The zero-inflated model (i.e., for commission error) does not estimate the residual variance, therefore, we calculated the latent intraclass correlation coefficient. The latent ICC uses the standard latent logit scale, which was set to 1. The 95% confidence intervals were not calculated for this model since reliable bootstrapping is not available for non-Gaussian models (Nakagawa et al., 2017).

## 3 Results

### 3.1 Participants

Seventeen participants (five males, twelve females) were included in the final analysis with a mean age of 67.76 (SD = 3.95). The sample was highly educated, the mean number of years being 17.41 (SD = 2.74), which is locally equivalent to a Bachelor's degree. The participants had MoCA scores greater than 26, with the exception of two participants: one did not complete the questionnaire and one participant had a score of 20 (note that this participant appeared anxious and distracted during MoCA testing, and the score was deemed an inaccurate representation of their current cognitive abilities—they were thus retained in the sample). Subjective sleep quality, as indicated by overall scores on the Pittsburgh Sleep Quality Index was 4.00 (SD = 3.35), indicating that our sample reported generally having good quality sleep, though with considerable variability (Buysse et al., 1989)—four participants reported having poor subjective sleep quality on the PSQI (score >5). Participants scored an average of 62.94 (SD = 9.53) on the MEQ, suggesting that the majority of the sample can be characterized by having a moderate morning chronotype. Participant responses ranged from intermediate to definite morning type. Demographic characteristics are listed in Table 1.

**Table 1:** Demographic characteristics and overall cognitive performance.

**Variable**	**Mean**	**SD**
**Demographic characteristics**		
Age	67.76	3.95
Education (years)	17.41	2.74
MEQ	62.94	9.53
PSQI	4.00	3.35
BDI-II	1.88	2.89
BAI	2.53	2.32
MoCA	27.38	2.31
**Cognitive performance**		
GNG RT (ms)	430.58	52.81
Commission error (%)	6.12	5.52
Hit (%)	94.16	19.09
Incongruent RT (ms)	816.53	145.84
Congruent RT (ms)	718.74	146.45
Congruent direction Acc (%)	99.70	1.27
Incongruent direction Acc (%)	97.50	3.20
Congruent location Acc (%)	99.88	0.56
Incongruent location Acc (%)	97.83	2.84
Simon effect Acc (%)	–2.11	2.18
Switch effect Acc (%)	0.23	1.38
Simon effect RT (ms)	97.79	49.08
Switch effect RT (ms)	–18.68	25.75

Cognitive performance scores represent group averages and standard deviations (SD) averaged across all usable nights for each participant. GNG, Go/NoGo; MEQ, Morningness-Eveningness Questionnaire; PSQI, Pittsburgh Sleep Quality Index; BDI-II, Beck Depression Inventory II; BAI, Beck Anxiety Inventory; MoCA, Montreal Cognitive Assessment; Acc, accuracy; RT, reaction time.

### 3.2 Behavioral results

#### 3.2.1 Inhibition

**Figure 3:**
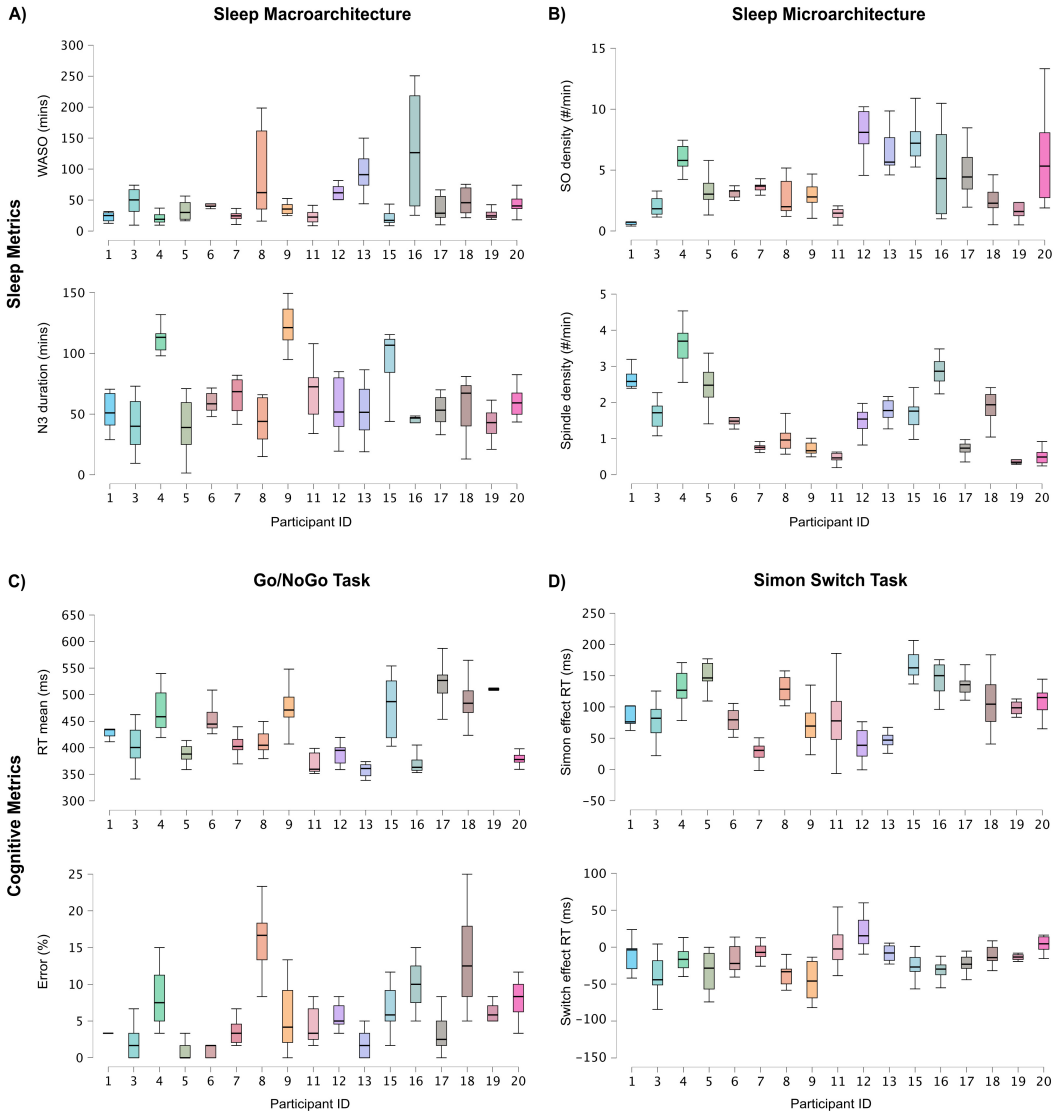
**(A)** Variability in WASO duration and N3 duration across participants and nights. **(B)** Variability in spindle density and slow oscillation density across participants and over nights. **(C)** Variability in reaction time (ms) and error percent on the Go/NoGo task across participants and days. **(D)** Variability in the Simon effect on reaction time and the switch effect on reaction time on the Simon switch task across participants and days. Participants 2, 10, and 14 were excluded from the figure and were omitted from our analysis. Each participant is represented by a color. The box represents the 25th to the 75th percentiles with the line being the median. The error bars represent 1.5 × the interquartile range. In all measures, considerable variability is observed: across participants (means), between nights within-participants, and in the distribution of values across nights and participants.

The Go/NoGo task was used to measure cognitive inhibition and sustained attention. The mean reaction time (averaged first across repeated measurements, for each participant) on the “go” trials was 430.58 ms (SD = 52.81) and the hit rate was 94.16% (SD = 19.09), suggesting that on average, participants remained actively engaged in the task throughout the study. The average commission error (i.e., incorrectly responding during a “no go” trial) was 6.12% (SD = 5.52). Response times and commission errors varied considerably across days (see Figure 3C).

#### 3.2.2 Cognitive flexibility

The Simon switch task was used to measure cognitive flexibility. The Simon effect refers to the increase in reaction time observed between congruent and incongruent trials. As expected, the mean Simon effect on accuracy was –2.11% (SD = 2.18), suggesting that accuracy was lower on the incongruent trials compared to congruent trials. The average reaction time on the Simon effect was 97.79 ms (SD = 49.08), demonstrating a longer reaction time on the incongruent trials, consistent with previous studies. The switch effect refers to the increase in reaction time observed on switch trials compared to non-switch trials. The switch effect on accuracy was 0.23% (SD = 1.38), suggesting that performance was better on non-switch trials compared to switch trials, which was expected. The switch effect on reaction time was –18.68 ms (SD = 25.75). Surprisingly, participants were faster on switch trials compared to non-switch trials.

### 3.3 EEG quality and multi-night in-home recording feasibility

To determine the feasibility of multi-night EEG recordings in older adults, we examined the percentage of usable data and signal quality sufficient for event detection. The number of usable nights per participant ranged from 4 to 15 (*M* = 10.47, SD = 3.94) after exclusions. Almost all participants were able to complete the entire 14-night protocol. Of the total 250 usable nights collected, 28.40% were excluded for poor quality or missing data (16 days or 6.40% were missing behavioral or sleep data). This percentage of data loss is in line with previous studies using the same headband in a lab setting (Ravindran et al., 2025). The average channel quality after removing poor quality channels was 70.96% (SD = 10.67), which is slightly lower than a previous study in older adults (Gabb et al., 2025). Based on the above, we found the data to be of sufficient quality to conduct an analysis of neural activity during sleep. Participants seemed to find the procedure relatively easy, and none reported having trouble sleeping using the device. They also expressed no difficulties completing the online cognitive tasks. We examined information from the daily sleep log to confirm that participants' general wellbeing was high during the study period (noting that one participant did not complete the sleep log). On a Likert scale from 1 to 5, participants reported feeling generally energetic (*M* = 4.12, SD = 0.85), concentrated (*M* = 4.37, SD = 0.86), well (*M* = 4.83, SD = 0.60), relaxed (*M* = 4.54, SD = 0.80), and in a good mood (*M* = 4.43, SD = 0.75), suggesting that their participation in this extended recording protocol was not perceived to be problematic. To ensure that the headband did not create differential effects across nights, for example, an initial disruption followed by habituation, we conducted an additional GLMM analysis with night as the fixed effect. To capture sleep disruptions, we used the index of sleep fragmentation (WASO) as the predictor and found no main effect of night [*F*_(1, 178)_ = –0.14, *p* = 0.82], also suggesting that subsequent observations of sleep variability were not caused by a process of habituation to the headband. To assess potential learning effects, we examined whether testing day predicted task performance using GLMMs with day as a fixed effect and participant as a random effect [e.g., model: RT ~ day + (1 ∣ subject)]. There were no significant effects of testing day on reaction time on the Go/NoGo, the switch effect, or on commission error percent (*p*> 0.05), indicating that learning effects did not strongly influence our results. There was a significant effect of day on reaction time on the Simon effect (*p* = 0.004). To account for this, the Simon effect model was conducted with and without day as a fixed effect, and since the results did not differ, the model without day is reported below.

### 3.4 Sleep quality

#### 3.4.1 Characterization of sleep metrics

Group mean and standard deviation for sleep characteristics (for each participant across all nights) is reported in Table 2. Focusing on metrics of sleep microarchitecture, the mean spindle density (spindles/min) was 1.52 (SD = 0.96), which is lower compared to what has been previous studies (e.g., Papalambros et al., 2017 reported a spindle density of 5.5). This difference may be due to differences in electrode montage. The high standard deviation and variability in spindle density has also been reported in previous work, for example Guadagni et al. (2021) reported a mean of 2.46 with a SD of 1.56. Slow oscillation (SO) density was 4.20 (SD = 2.85) and is also lower than what was found in previous studies, for example Lafrenière et al. (2023) reported a SO density of 6.0 using a frontal electrode. Nonetheless, detected spindles and slow oscillations indicate clear, characteristic forms in group averages, suggesting that the measurement and detection techniques are sufficient to address the main research questions (see Figure 2).

**Table 2:** Mean sleep characteristics across participants.

**Variable**	**Mean**	**SD**
**Sleep characteristics**		
N1 duration (min)	34.60	11.30
N2 duration (min)	212.04	59.02
N3 duration (min)	64.22	31.79
REM duration (min)	106.50	43.89
N1 percentage (%)	8.44	3.06
N2 percentage (%)	50.40	10.89
N3 percentage (%)	15.53	7.70
REM percentage (%)	25.62	10.33
WASO (min)	48.11	41.03
TST (min)	417.36	65.12
Number awakenings	24.20	7.58
Sleep efficiency (%)	83.98	9.22
SO density	4.20	2.85
SO amplitude (μV)	86.48	6.50
Spindle density	1.52	0.96
Spindle duration (s)	0.66	0.11
Spindle amplitude (μV)	6.86	1.68

WASO, wake after sleep onset; TST, total sleep time; SO, slow oscillations; SD, standard deviation.

#### 3.4.2 Within-participant vs. between-participant variability

The means and standard deviations of sleep architecture are reported in Table 2. To characterize general sleep quality, we examined variability in WASO and N3 duration. The mean number of minutes spent in wake after sleep onset (WASO) was 48.11 (SD = 41.03). Visual inspection suggests high interindividual variability (see Figure 3A). On average, participants spent 64.22 min in N3 or deep sleep, with considerable differences between participants (SD = 31.79; see Figure 3A). This pattern of results demonstrates high intra- and interindividual variability, suggesting that the multi-night datasets capture nightly changes in sleep quality, and conversely, that a single night of data may be an inadequate measure of an individual's overall sleep quality for many participants. Note that within-participant sleep quality variability has been suggested to negatively impact cognitive performance in older adults and will thus be investigated further as an exploratory analysis, below.

### 3.5 Relationship between sleep and cognition

#### 3.5.1 Sleep quality and cognitive performance

**Figure 4:**
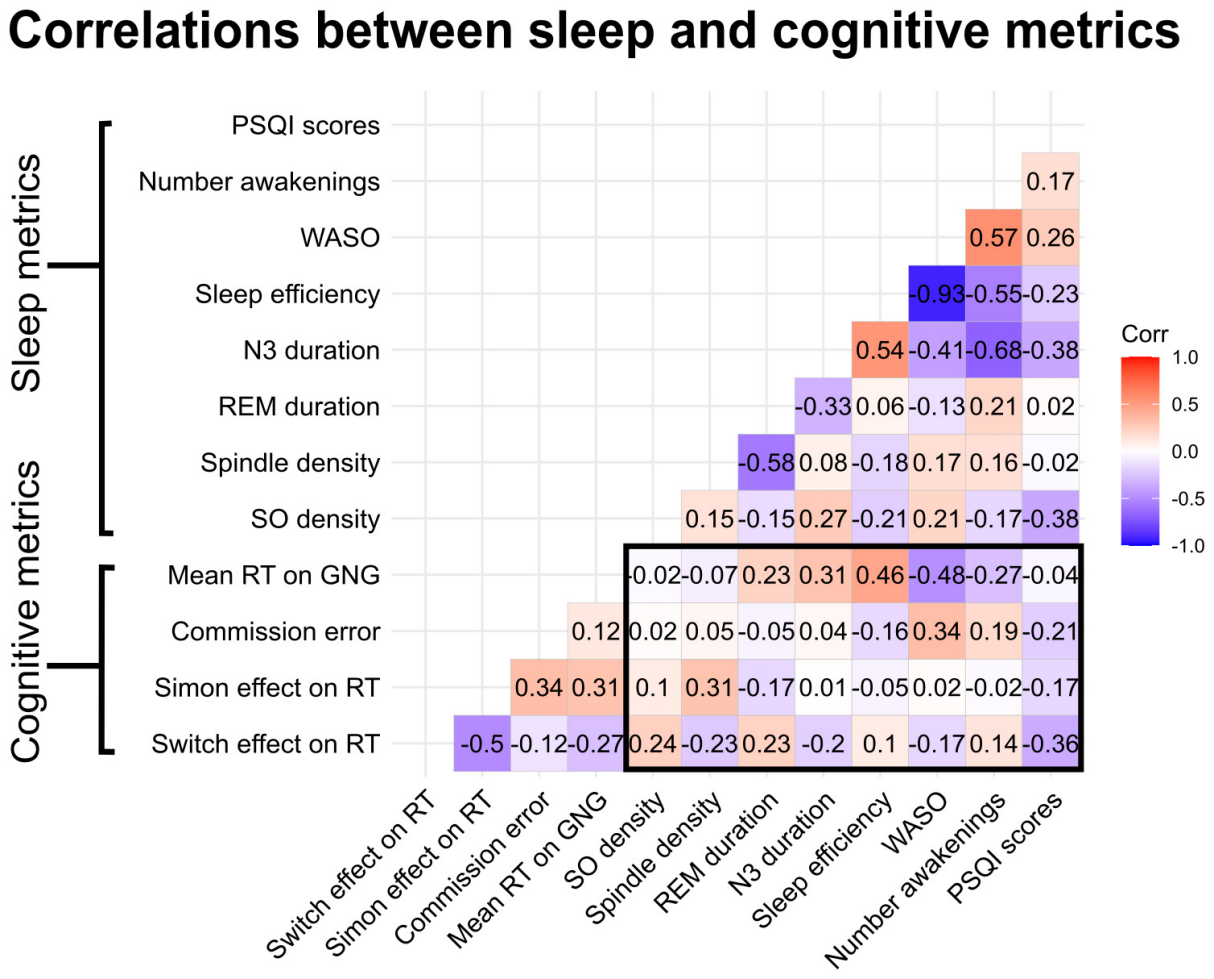
Correlations between mean sleep metrics of interest and mean cognitive performance. Blue coloring indicates negative and red coloring indicates positive relationships. Light shading indicates little or no association between two variables. The main correlations of interest are between the cognitive and sleep variables (in the outlined box); none were statistically significant.

To assess the relationship between general sleep characteristics and cognitive performance, we first performed correlational analyses on mean metrics across all available nights for each participant. This analysis was performed on the basis that multiple measurements would be more representative of overall sleep and performance than would be acquired during a single night. We examined the relationship between variables of interest (spindle density, slow oscillation density, WASO, and N3) and performed exploratory correlations with other measures of sleep quality, including PSQI scores. We found no associations between any of our sleep quality metrics, including WASO and the number of minutes spent in N3 (see Figure 4 for all correlations). Specifically as regards our main hypotheses, we found no association between reaction time on the Go/NoGo task and mean spindle density (*r* = –0.07, *p* = 0.96) or slow oscillation density (*r* = –0.02, *p* = 0.96). Similarly, we found no association between commission error and spindle density (*r* = 0.05, *p* = 0.96) or slow oscillation density (*r* = 0.02, *p* = 0.96). These results suggest that sleep microarchitecture and inhibition may be relatively independent in older adults. In terms of the Simon effect on reaction time, there was no association between spindle (*r* = 0.31, *p* = 0.92) or slow oscillation density (*r* = 0.10, *p* = 0.96). We also found no association between spindle density (*r* = –0.23, *p* = 0.92) or slow oscillation density (*r* = 0.24, *p* = 0.92) and switch effect on reaction time. These results suggest that there may not be a strong association between spindle and slow oscillation density and cognitive flexibility in older adults, either.

Given the intraindividual variability in microarchitecture observed in our study, which is consistent with previous findings (Chen et al., 2025), as well as evidence suggesting that sleep variability influences cognitive performance (Kuan et al., 2022), we performed exploratory correlational analyses to investigate whether intraindividual variability in sleep metrics (i.e., standard deviation in scores across nights) was correlated with cognitive performance. We found no association between WASO or deep sleep variability and cognitive performance (see Supplementary Table 1 for all correlations; reported *p*-values are uncorrected). We found that intraindividual slow oscillation density variability was not significantly correlated with reaction time (*r*s = 0.04, *p* = 0.81), commission error (*r*s = 0.42, *p* = 0.56), Simon effect on reaction time (*r*s = 0.30, *p* = 0.73), or switch effect on reaction time (*r*s = −0.11, *p* = 0.72). Similarly, we found no association between sleep spindle density variability and reaction time on the Go/NoGo (*r*s = −0.16, *p* = 0.81), commission error (*r*s = 0.35, *p* = 0.66), switch effect on reaction time (*r*s = −0.33, *p* = 0.66), or the Simon effect on reaction time (*r*s = 0.49, *p* = 0.56). These results also suggest that variability within individual microarchitecture itself is not correlated with inhibition or cognitive flexibility in older adults.

#### 3.5.2 Nightly sleep quality and next-day cognitive performance

To investigate whether nightly sleep quality influences next-day cognitive performance, we performed GLMM analyses. To inform future research study designs, we calculated intraclass correlation coefficients (ICCs) for all models, as few studies have reported these essential parameters for sleep and cognition research in older adults. We used slow oscillation and spindle density to examine microarchitecture and WASO for general sleep quality, as it has been associated with cognitive performance (Kuan et al., 2022; Wilckens et al., 2014). Previous literature has shown sex differences in sleep microarchitecture (Carrier et al., 2011; Martin et al., 2013), therefore, we ran the models with and without sex as a variable. We found no significant effect of sex and present the models without sex. Spindle density, slow oscillation density, and WASO were included as fixed effects and subject was included as a random effect. We first investigated reaction time on the Go/NoGo [RT ~1 + SO density + spindle density + WASO + (1 ∣ subject)]. We found no main effect of slow oscillation density, spindle density, or WASO on reaction time (see Table 3 for complete model results). Subsequently, we performed a Type III ANOVA and found that slow oscillation density [*F*_(1, 170)_ = 2.29, *p* = 0.13], spindle density [*F*_(1, 170)_ = 0.29, *p* = 0.58], and WASO [*F*_(1, 170)_ = 0.20, *p* = 0.66] did not significantly explain variability in reaction time. Based on the GLMM variance components, we calculated the ICC. We found a high ICC of 0.64 (95% CI: [0.41, 0.77]), which would suggest that 64% of the variance observed in reaction time can be attributed to differences between-participants.

**Table 3:** Model components for the generalized linear mixed models and variance components for the random effects (reported in full to inform the design of future multi-night studies).

**Model**	**Predictor**	**β**	**SE**	***Z***	***p***	**β (std.)**	**95% CI**
Reaction time	SO density	–2.10	1.39	–1.51	0.13	–0.10	[–0.39, 0.38]
	Spindle density	–3.83	7.06	–0.54	0.59	–0.06	[–0.22, 0.03]
	WASO	–0.04	0.09	–0.44	0.66	–0.03	[–0.14, 0.09]
Random effects variance	Subject (between)						2,404.27
	Residual (within)						1,440.80
Commission error	SO density	0.03	0.02	1.37	0.17	0.07	[–0.03, 0.18]
	Spindle density	0.07	0.09	0.77	0.44	0.06	[–0.10, 0.23]
	WASO	0.001	0.001	0.62	0.54	0.03	[–0.06, 0.12]
Random effects variance	Subject (between)						0.21
	Residual (within)						2,696.72
Simon effect	SO density	–0.09	1.08	–0.08	0.93	–0.001	[–0.13, 0.12]
	Spindle density	6.74	5.49	1.16	0.25	0.12	[–0.09, 0.33]
	WASO	0.09	0.07	1.36	0.18	0.08	[–0.03, 0.19]
Random effects variance	Subject (between)						2,404.27
	Residual (within)						1,440.80
Simon switch effect	SO density	–0.09	0.73	–0.13	0.89	–0.01	[–0.17, 0.15]
	Spindle density	0.42	3.22	0.13	0.89	0.02	[–0.22, 0.25]
	WASO	–0.04	0.05	–0.88	0.37	–0.07	[–0.21, 0.08]
Random effects variance	Subject (between)						1,383.31
	Residual (within)						869.35

SO, slow oscillation; WASO, wake after sleep onset; Std, standardized; CI, confidence interval.

For commission error and sleep metrics (Commission error ~1 + SO density + spindle density + WASO + (1 ∣ subject), family = beta family, *zi* = ~1), we found no main effect of slow oscillation density, spindle density, or WASO on commission error. We also found that slow oscillation density [*F*_(1, inf)_ = 1.88, *p* = 0.17], spindle density [*F*_(1, *inf*)_ = 0.59, *p* = 0.44], and WASO [*F*_(1, *inf*)_ = 0.38, *p* = 0.54] did not significantly explain variability in commission error. These results suggest that spindle and slow oscillation density and WASO do not influence next-day performance on a task measuring inhibition in older adults. The latent ICC was calculated for the commission error model, and we found a low correlation of 0.18, meaning 18% of the total variance is explained by between-participant differences, suggesting that within-participant differences account for the majority of the variance observed in commission errors.

Finally, we explored the relationship between sleep quality and cognitive flexibility. The Simon effect on reaction time model [Simon effect RT ~1 + SO density + spindle density + WASO + (1 ∣ subject)], revealed no main effect of slow oscillation density, spindle density, nor WASO on the Simon effect on reaction time. We also found that slow oscillation [*F*_(1, 171)_ = 0.007, *p* = 0.93], spindle density [*F*_(1, 171)_ = 1.34, *p* = 0.25], and WASO [*F*_(1, 171)_ = 1.84, *p* = 0.18] did not significantly explain variability in the Simon effect on reaction time. We calculated the ICC from our GLM model. We found a high ICC of 0.63 (95% CI: [0.39, 0.77]), which indicates that 63% of the variance observed in reaction time can be attributed to differences between-participants. When investigating the switch effect on reaction time [Switch effect RT ~1 + SO density + spindle density + WASO + (1 ∣ subject)], we did not find a main effect of slow oscillation density, spindle density, or WASO. We performed a Type III ANOVA and found that sleep spindle density [*F*_(1, 171)_ = 0.01, *p* = 0.93], slow oscillation density [*F*_(1, 171)_ = 0.03, *p* = 0.87], and WASO [*F*_(1, 171)_ = 0.775, *p* = 0.38] did not account for variability in the switch effect on reaction time. These results would suggest that spindle and slow oscillation density and WASO do not clearly influence next-day performance on tasks measuring cognitive flexibility in older adults. Finally, we calculated the ICC for this model and found a low ICC of 0.35 (95% CI: [0.14, 0.55]). This result suggests that 35% of the variance observed in reaction time can be attributed to between-participant differences.

## 4 Discussion

The purposes of this study were to demonstrate the feasibility of multi-night in-home studies in older adults, to quantify variability of sleep quality metrics across nights and examine relationships between both average sleep quality and sleep variability with cognitive performance, and to explore the effect of nightly sleep quality on next-day cognitive performance. Additionally, we aimed to provide essential variance components and effect size estimates to guide future study planning.

We demonstrated that measuring EEG using a multi-night, in-home study design in older adults is feasible. Participants reported that the in-lab practice session provided sufficient familiarity with the experimental procedures and equipment, and did not describe difficulties following instructions at home. Although we expected a relatively high proportion of data loss as compared with studies in which equipment is applied by trained research personnel, the amount of usable data was equivalent to previous studies using the same device in a laboratory setting in older adults (i.e., 71% as compared with 73%; Ravindran et al., 2025). Additionally, after preprocessing the EEG data, we determined that the data were of acceptable quality to extract microarchitectural sleep features (Figure 2). These results support the use of EEG data collection in older adults over many nights, in an ecologically valid and naturalistic setting. Furthermore, participants' high accuracy scores on the tasks throughout the study indicate stable study engagement over the two week testing period, further supporting the feasibility of multi-night in-home study designs.

The characterization of sleep metrics across participants and nights revealed considerable variability in sleep macro- (i.e., WASO and N3) and microarchitecture (i.e., spindles and slow oscillations), both between participants and across nights on our main variables of interest: time spent in N3 (deep sleep) and WASO (Figure 3). These findings are consistent with previous work using subjective self-report measures of sleep quality that demonstrate nightly fluctuations in WASO over 14 nights in older adults (Shoji et al., 2015). Our study extends these findings by using objective EEG sleep measures, which allowed us to capture microarchitecture over many nights. We also found high nightly variability in sleep spindle and slow oscillation density (as well as differences between participants in how variable nights were), as reported previously in older adults (Gomez-Pilar et al., 2020; Chen et al., 2025). In addition to the variability in sleep quality metrics, we observed variability in performance on the Go/NoGo and Simon switch tasks across multiple days (Figure 3).

After averaging metrics across multiple nights for each participant, we found no associations between sleep characteristics and performance on the two cognitive tasks. This observation contrasts with some previous studies, for example those suggesting that increased WASO is associated with poorer cognition (Blackwell et al., 2011; Yaffe et al., 2014), and with findings showing that sleep spindles are associated with visual attention in older adults (Lafortune et al., 2014). We also unexpectedly found a faster reaction time on non-switch trials compared to switch trials on the Simon switch task. This may have been due to task interference as a result of dual instructions, meaning that conflicting potential responses could have inadvertently increased the cognitive load on non-switch trials, as has been observed in a previous study using a task switching paradigm (Wylie and Allport, 2000). This result suggests that there may have been a higher cognitive load attributed with non-switch trials which may have impacted the switch effect as it relates to sleep metrics. In general, there is mixed evidence in the literature regarding the effect of sleep quality on cognitive performance depending on the sleep and cognitive measures used. For example, one study found that WASO was not associated with reasoning but was associated with faster reaction time in older adults (McCrae et al., 2012) and another study found that mean sleep metrics, including WASO, were not associated with global cognitive impairment or verbal memory (Kuan et al., 2022). These mixed findings highlight the complexity of sleep-cognition relationships and suggest they may be sensitive to the specific cognitive measures used.

Despite the variability observed in both macro- and microarchitecture and in cognition, our exploratory analyses revealed no associations between any of the sleep metrics standard deviations and average cognitive performance. These results suggest that within-participant sleep quality variability may not be a strong predictor of inhibition and cognitive flexibility. Furthermore, this result has methodological implications: the nightly differences observed in both sleep metrics and measures of performance suggest that, at least in older adults, study designs measuring only a signal or a small number of time points might not reflect a participants' overall pattern of sleep quality or cognitive ability. Our findings illustrate the value of multi-night designs to characterize sleep and cognition in the aging population, and emphasizes the need for a more comprehensive investigation of potential effects of sleep quality on cognition.

Finally, we assessed whether nightly sleep quality variability influences next-day cognitive performance. Contrary to our hypothesis, our results revealed that spindle density, slow oscillation density, and WASO did not drive next-day cognitive performance. One explanation for the lack of associations between cognitive performance and our measures of sleep quality is that older adults employ a compensatory mechanism that buffers potential negative effect of poor sleep on next-day performance. Previous studies have shown that older adults are less affected by sleep loss as compared to young adults in terms of their performance on attentional tasks (Duffy et al., 2009; Sagaspe et al., 2012), which may be why we did not see clear cognitive effects as a result of nightly sleep quality differences. According to the Compensation-Related Utilization of Neural Circuits Hypothesis (CRUNCH), older adults show increased activity in the PFC to support the demands of cognitive tasks (Reuter-Lorenz and Cappell, 2008). An fMRI study provided support for this idea, showing that while older and younger adults performed similarly, older adults had greater blood oxygen-level dependent (BOLD) activity in certain regions of the prefrontal cortex (PFC) following sleep deprivation (Almklov et al., 2015), suggesting that over-activation of regions in the PFC may be functioning as a compensatory mechanism to account for sleep loss. Interestingly, increased activation in regions of the PFC has also been observed in young adults who show better performance on inhibitory tasks compared to young adults who did not show this increase (Chuah et al., 2006). This PFC-based compensatory mechanism may explain the relative resilience to changes in sleep quality observed in our study. Another explanation for the lack of association between sleep quality and cognition is that these effects may not be as evident in healthy populations compared to clinical populations. Seelye et al. (2015) found that after controlling for variables such as pain, low mood, and medication use, the relationship between sleep disturbance and cognitive functioning in older adults was no longer significant. Small fluctuations in sleep may not be strongly related with cognitive functioning in an overall healthy sample.

Despite the lack of association between sleep variables and next-day cognitive performance, we report standardized effect sizes and variance components for each model to inform future research study designs. Our results revealed relatively small standardized effect sizes (0.03–0.12), suggesting small effects of our chosen sleep metrics on cognitive performance. Additionally, we performed intraclass correlation coefficients for each GLM model. Our models yielded generally high ICCs (~0.60) for reaction time and the Simon effect on reaction time, suggesting greater between- than within-participant differences. These parameters, which were not previously available for multi-night sleep-cognition studies in older adults, suggest that for these two variables, a larger sample size with fewer repeated measurements per participant is recommended. In contrast, commission error and the switch effect on reaction time yielded relative low ICCs (0.18 and 0.35). For commission error and the switch effect, a higher number of repeated measures is warranted given the high within-participant variances. The complete variance components and effect size estimates presented in Table 3 are provided to enable researchers to use power analysis tools for optimizing the trade-offs between sample size and the number of measurements, thereby facilitating appropriately powered and replicable future research in this area.

While the present work contributes an empirical investigation of sleep quality and next-day cognitive performance, the study has several limitations, notably the relatively small sample size. Although the repeated measures is a relative strength and allows us to examine the main research questions, we initially planned for a larger sample size to maximize sensitivity in exploratory analyses to statistical relationships with additional variables, such as PSQI scores and other sleep characteristics. Due to disruptions of service with the commercial EEG device, we were only able to collect a sample of 20 participants. Our study supports the need for open science and cost-effective tools as alternatives both to traditional polysomnography and to commercial solutions. New community-driven portable devices, such as the Portiloop (Valenchon et al., 2022), are promising tools that aim to increase accessibility of equipment to sleep researchers, ensure continuity of data collection throughout studies, and ultimately advance our understanding of relationships between sleep and cognition. Although we collected many nights worth of EEG and cognitive data (179 recordings), and were able to conduct a preliminary investigation using GLMMs, the high variability within these measures suggests that a larger sample size is needed. *Post-hoc* power simulations indicated that our design was adequately powered to detect large effects (β ≈ –5) but had limited sensitivity (11%–50% power) for smaller effects. Our observed standardized effects were small (β ≤ 0.12), thus we cannot rule out that subtle sleep-cognition relationships exist but were undetectable with our sample. Additionally, our sample was predominantly female (12 of 17 participants), which is consistent with volunteer bias patterns observed in community-recruited studies of older adults but may limit generalisability. Future work with larger and more balanced samples will be better able to examine potential sex differences in sleep-cognition relationships, which is particularly relevant given documented sex differences in sleep microarchitecture (Carrier et al., 2011; Martin et al., 2013).

Another limitation concerns the choice of cognitive tasks. We selected these two tasks because they are widely used measures of inhibition and cognitive flexibility and have shown some susceptibility to poor sleep quality (e.g., Wilckens et al., 2014; Fang et al., 2022). Our participants performed quite well on both tasks across days, suggesting these particular executive functions may be resilient to the sleep variations we measured. Additionally, certain design choices may have reduced task difficulty: the Go/NoGo task used dual cues (color and text) to ensure accessibility for unsupervised at-home administration, which likely made stimulus discrimination easier than single-cue designs. More demanding paradigms, such as the Stop-Signal Task (Verbruggen and Logan, 2008), or adaptive procedures that adjust difficulty based on performance, might reveal sleep-related effects that our tasks could not detect. It also remains to be seen if other cognitive functions might be more affected by nightly fluctuations in sleep quality. Furthermore, given our sample size, we focused specifically on slow oscillation and spindle density and WASO in relation to these executive tasks, but future research should explore other sleep metrics and cognitive functions. For example, memory consolidation and other more complex executive functions may show stronger relationships with sleep variability than inhibition and cognitive flexibility.

A consideration for the interpretation of our results concerns spindle detection methodology in older adults. Although previous findings suggest that spindle density is highly variable in older adults (Guadagni et al., 2021), the challenges of consistent spindle detection across individuals may have contributed to our null findings. Older adults exhibit more variable and weaker sleep spindles, and our choice of detection algorithm, electrode montage, and parameters may have worked better for some participants than others. The removal of one parameter from the Lacourse algorithm due to our different EEG montage was intended to improve sensitivity, but this approach may have reduced detection accuracy for individuals with particularly low-amplitude spindles or high background activity. Future work should explore optimal methods for consistent spindle detection across older adults with varying spindle characteristics. Personalized spindle detection approaches that adapt to individual neural characteristics may offer a solution to these challenges (Sobral et al., 2025). Such methods could improve the reliability of spindle-cognition relationships by accounting for inter-individual differences in spindle morphology. Future studies should investigate other spindle characteristics that might show stronger cognitive associations, such as frequency, duration, and topographical distribution (Purcell et al., 2017). Additionally, rather than using a fixed electrode derivation across all participants and nights, we selected the channel with the best signal quality from among frontal-occipital derivations. While this maximized data retention, it may have introduced variability across recordings. However, given that electrodes within each cluster were within a few centimeters of one another and spindle topographies are broadly distributed (see Figure 2 in Campos-Beltrán and Marshall, 2021), we do not expect this to have substantially affected our results. Previous studies have demonstrated that spindle power is strongest at central electrodes such as Cz (Dehghani et al., 2011), and that fast spindles predominate centrally while slow spindles are more prominent frontally (Campos-Beltrán and Marshall, 2021). Our frontal-occipital derivations would have captured both fast and slow spindles, though possibly with a bias toward slow spindles compared to more commonly used Cz-mastoid derivations. However, frontal derivations may be particularly relevant for aging research, as age-related reductions in spindle amplitude and density are most pronounced in anterior regions (Martin et al., 2013).

Our sample was highly educated and high-functioning, which may explain our null findings. We intentionally studied relatively young older adults (60–75 years) who were comfortable with technology, as sleep disturbance may be an early indicator of cognitive decline (Zawar et al., 2023), and early identification could enable more effective interventions with greater impact on wellbeing. However, this high-functioning population may actively manage age-related changes, potentially masking sleep-cognition relationships that would be apparent in more cognitively vulnerable groups. We did not formally assess sleep disorders and thus this might have affected the results of our study given that there are known decreases in task performance in those with sleep apnea and insomnia (de Souza Bezerra et al., 2024; Fang et al., 2022). Future work should examine more diverse samples, including older adults with varying cognitive reserve and sleep disorders, to better characterize when and in whom sleep-cognition relationships emerge, namely whether sleep-cognition relationships are stronger or more detectable when sleep disturbance is more severe. Comparing individuals with clinical insomnia to those with suboptimal but non-clinical sleep quality could help clarify the threshold at which sleep disturbances begin to measurably affect executive function.

The present study demonstrates that older adults can successfully collect high-quality EEG and cognitive data in their own homes across multiple nights, opening new possibilities for naturalistic sleep research in aging populations. Using portable devices, we captured the substantial variability in sleep that characterizes real-world sleep in older adults – variability that would be missed in traditional single-night laboratory studies. Surprisingly, despite clear fluctuations in both sleep macro- and microarchitecture and in executive functions (i.e., inhibition and cognitive flexibility), we did not find relationships between average sleep quality and next-day cognitive performance. Neither average sleep quality nor within-participant sleep variability predicted cognitive performance, suggesting these executive functions may be resilient to natural sleep fluctuations in healthy older adults. Despite these null findings, we provide effect sizes and variance components to inform future study designs. The results suggest that larger sample sizes may be needed to detect certain relationships between sleep and cognitive performance. As portable sleep technologies continue to evolve, multi-night home-based studies will be essential for understanding how sleep-cognition relationships unfold in real-world settings. The methodological parameters provided here will enable researchers to design appropriately powered studies, ultimately informing more effective interventions to support cognitive health throughout the lifespan.

## Data Availability

The datasets presented in this study can be found in online repositories. The names of the repository/repositories and accession number(s) can be found at: https://osf.io/rhxcv/?view_only=e32bdctac4bd4725ae6638a0b84a4e7f.

## Footnotes

1
https://pavlovia.org
