# Supplementary material for: Nightly variations in sleep quality and next-day cognitive performance: an in-home study in healthy older adults
Source: Front Aging Neurosci. 2026 Mar 9;18:1714063. doi: 10.3389/fnagi.2026.1714063 (PMC13006850; doi:10.3389/fnagi.2026.1714063)
Supplement: Supplementary file 1 [file Data_Sheet_1.pdf]

Supplementary Material

Spearman's Correlations

Variable		RT mean	Error percent	Switch effect RT	Simon effect RT	SO density SD	Spindle density SD	N3 duration SD	WASO SD
1. RT mean	Spearman's rho	—							
	p-value	—							
2. Error percent	Spearman's rho	0.20	—						
	p-value	0.44	—						
3. S witch effect RT	Spearman's rho	-0.28	-0.05	—					
	p-value	0.28	0.86	—					
4. Simon effect RT	Spearman's rho	0.23	0.35	-0.42	—				
	p-value	0.38	0.17	0.10	—				
5. SO density SD	Spearman's rho	0.04	0.42	-0.11	0.30	—			
	p-value	0.88	0.10	0.67	0.25	—			
6. SP density SD	Spearman's rho	-0.16	0.35	-0.33	0.49*	0.19	—		
	p-value	0.55	0.17	0.20	0.05	0.46	—		
7. N3 duration SD	Spearman's rho	-0.19	-0.04	0.29	-0.04	-0.21	0.12	—	
	p-value	0.47	0.87	0.26	0.89	0.42	0.65	—	
8. WASO SD	Spearman's rho	-0.41	0.22	-0.13	0.08	0.39	0.31	-0.13	—
	p-value	0.10	0.39	0.63	0.77	0.13	0.22	0.61	—

* $p < .05$, ** $p < .01$, *** $p < .001$

Supplementary Table 1. Table with the Spearman correlations between the standard deviation of sleep metrics and performance on the two cognitive tasks. Listed here are the uncorrected p-values.
